# Feasibility of artificial intelligence–supported assessment of bone marrow infiltration using dual-energy computed tomography in patients with evidence of monoclonal protein — a retrospective observational study

**DOI:** 10.1007/s00330-021-08419-2

**Published:** 2021-12-18

**Authors:** Philipp Fervers, Florian Fervers, Jonathan Kottlors, Philipp Lohneis, Philip Pollman-Schweckhorst, Hasan Zaytoun, Miriam Rinneburger, David Maintz, Nils Große Hokamp

**Affiliations:** 1 Department of Diagnostic and Interventional Radiology, University Hospital Cologne, Kerpener Straße 62, 50937 Cologne, Germany; 2 System Technologies and Image Exploitation IOSB, Fraunhofer Institute of Optronics, Fraunhoferstraße 1, 76131 Karlsruhe, Germany; 3 Institute of Pathology, University Hospital Cologne, Kerpener Straße 62, 50937 Cologne, Germany; 4 Chair in Marketing Science and Analytics, University of Cologne, Albertus-Magnus-Platz, 50923 Cologne, Germany

**Keywords:** Multiple myeloma, Monoclonal gammopathy of undetermined significance, Neural networks, computer, Tomography, X-ray computed, Bone marrow

## Abstract

**Objectives:**

To demonstrate the feasibility of an automated, non-invasive approach to estimate bone marrow (BM) infiltration of multiple myeloma (MM) by dual-energy computed tomography (DECT) after virtual non-calcium (VNCa) post-processing.

**Methods:**

Individuals with MM and monoclonal gammopathy of unknown significance (MGUS) with concurrent DECT and BM biopsy between May 2018 and July 2020 were included in this retrospective observational study. Two pathologists and three radiologists reported BM infiltration and presence of osteolytic bone lesions, respectively. Bone mineral density (BMD) was quantified CT-based by a CE-certified software. Automated spine segmentation was implemented by a pre-trained convolutional neural network. The non-fatty portion of BM was defined as voxels > 0 HU in VNCa. For statistical assessment, multivariate regression and receiver operating characteristic (ROC) were conducted.

**Results:**

Thirty-five patients (mean age 65 ± 12 years; 18 female) were evaluated. The non-fatty portion of BM significantly predicted BM infiltration after adjusting for the covariable BMD (*p* = 0.007, *r* = 0.46). A non-fatty portion of BM > 0.93% could anticipate osteolytic lesions and the clinical diagnosis of MM with an area under the ROC curve of 0.70 [0.49–0.90] and 0.71 [0.54–0.89], respectively. Our approach identified MM-patients without osteolytic lesions on conventional CT with a sensitivity and specificity of 0.63 and 0.71, respectively.

**Conclusions:**

Automated, AI-supported attenuation assessment of the spine in DECT VNCa is feasible to predict BM infiltration in MM. Further, the proposed method might allow for pre-selecting patients with higher pre-test probability of osteolytic bone lesions and support the clinical diagnosis of MM without pathognomonic lesions on conventional CT.

**Key Points:**

• *The retrospective study provides an automated approach for quantification of the non-fatty portion of bone marrow, based on AI-supported spine segmentation and virtual non-calcium dual-energy CT data.*

• *An increasing non-fatty portion of bone marrow is associated with a higher infiltration determined by invasive biopsy after adjusting for bone mineral density as a control variable (p* = *0.007, r* = *0.46).*

• *The non-fatty portion of bone marrow might support the clinical diagnosis of multiple myeloma when conventional CT images are negative (sensitivity 0.63, specificity 0.71).*

**Supplementary Information:**

The online version contains supplementary material available at 10.1007/s00330-021-08419-2.

## Introduction

Multiple myeloma (MM) and its precursor conditions smoldering myeloma and monoclonal gammopathy of unknown significance (MGUS) outline a continuous spectrum of monoclonal plasma cell disorders. Most cases of MM are preceded by the asymptomatic, premalignant disorders smoldering myeloma or MGUS, which are commonly diagnosed incidentally in patients presenting with other conditions [1]. MM is the second most common hematologic malignancy and primarily manifests in elderly patients (median age at diagnosis 66–70 years, age-standardized incidence 5 cases per 100,000 in the Western World) [2]. Standard work-up for diagnosis of plasma cell disorders includes laboratory testing, bone marrow (BM) biopsy of the iliac crest, and whole body imaging [3]. BM biopsy is required to evaluate the degree of plasma cell infiltration, while diagnostic imaging is employed to detect myeloma defining bone lesions [3].

Discrimination of MM against its premalignant conditions is crucial for patient management and prognosis, since MM obligates for a specific therapy. Vice versa, according to the latest recommendations, MGUS is managed in a “watch-and-wait” strategy [1, 4, 5]. Diagnosis of MM by International Myeloma Working Group (IMWG) guidelines demands a BM biopsy in almost all cases [3]. In contrast to the other obligatory procedures, BM biopsy still is a painful and uncomfortable experience for most patients [6, 7]. Despite IMWG recommendations, a recent large-scale clinical analysis challenged the obligation of regular invasive BM diagnostic for patients with evidence of monoclonal antibody, since in most cases it did not contribute to the diagnosis [5].

In dual-energy computed tomography (DECT), two imaging datasets with different energy spectra are achieved during a single acquisition. The disparity of attenuation between both datasets enables post-processing of DECT images with virtual removal of certain materials. This method is especially effective for the removal of materials with high atomic numbers, e.g. calcium, and subsequent creation of virtual non-calcium images (VNCa) [8, 9]. VNCa post-processing is based on a three-compartment model, which constitutes the total attenuation of the BM in non-contrast-enhanced CT images to fat, soft tissue, and bone mineral [10]. By virtual removal of the bone mineral content, the fatty and soft tissue portion of BM attenuation can be estimated [9, 11]. By applying this technique, DECT VNCa imaging yielded a similar performance for detection of MM lesions as compared to the gold standard MRI [9, 12–14] and achieved an excellent prediction of metabolic activity, when compared to the benchmark PET/CT [15].

In line with recent clinical studies questioning obligatory initial BM biopsy, and considering the promising recent results of first reports on VNCa imaging for the assessment of MM, our study had two objectives: first, to demonstrate the feasibility of artificial intelligence (AI) supported, automated assessment of VNCa data to investigate its association to BM infiltration by pathology results. The second objective was to explore cutoffs based on the quantitative analysis of BM attenuation in VNCa images for the presence of osteolytic lesions, as determined by radiology report of conventional CT images, and the clinical diagnosis of MM.

## Materials and methods

All procedures performed in studies involving human participants were conducted in accordance with the ethical standards of the institutional (number 20–1480) and national research committee and with the 1964 Helsinki declaration and its later amendments or comparable ethical standards. Informed consent was waived due to retrospective study characteristics.

### Patient enrollment

Inclusion criteria to our study comprised:

1. Whole-body low-dose CT according to the IMWG specified imaging protocol and concurrent diagnostic BM biopsy,
2. No history of specific therapy for MM to the day of CT and BM biopsy,
3. Evidence of monoclonal protein,
4. Imaging between May 2018 and July 2020,
5. Patient age > 18 years.

Exclusion criteria were:

1. History of secondary malignoma with requirement for specific therapy or bone involvement (*n* = 3),
2. Metal implants in the spine (*n* = 2).

### Assessment of clinical data

The quantitative infiltration rate of BM biopsies as per pathology report was noted. Each patient was assigned to the MGUS, smoldering myeloma and MM group based on IMWG recommendations.

### DECT image acquisition

All scans were performed on a commercially available spectral detector DECT scanner (IQon Spectral CT, Philips Healthcare), following the most recent recommendations of the IMWG [16]. Scans were unenhanced. Scan parameters were as follows: tube voltage 120 kV; tube current 70 mAs; collimation 32 × 0.625 mm; pitch 0.908; volumetric computed tomography dose index 7.4 mGy. Mean dose length product was 1091.7 ± 230.5 mGy*cm.

### DECT image reconstruction

All images were constructed in a 512 × 512 matrix, slice thickness 2 mm with an overlap of 1 mm. VNCa images were created by the vendor’s software in order to simulate each voxel’s attenuation in Hounsfield units (HU) without the calcium-specific contribution (IntelliSpace Portal, Spectral Diagnostics Suite, Philips Healthcare) [17]. In our study, calcium suppressed images were calculated with a high suppression index (index 25), as suggested by earlier results [15]. Detailed information about VNCa imaging has been provided in earlier studies [8, 18].

### Segmentation of the BM and assessment of DECT data

**Fig. 1:**
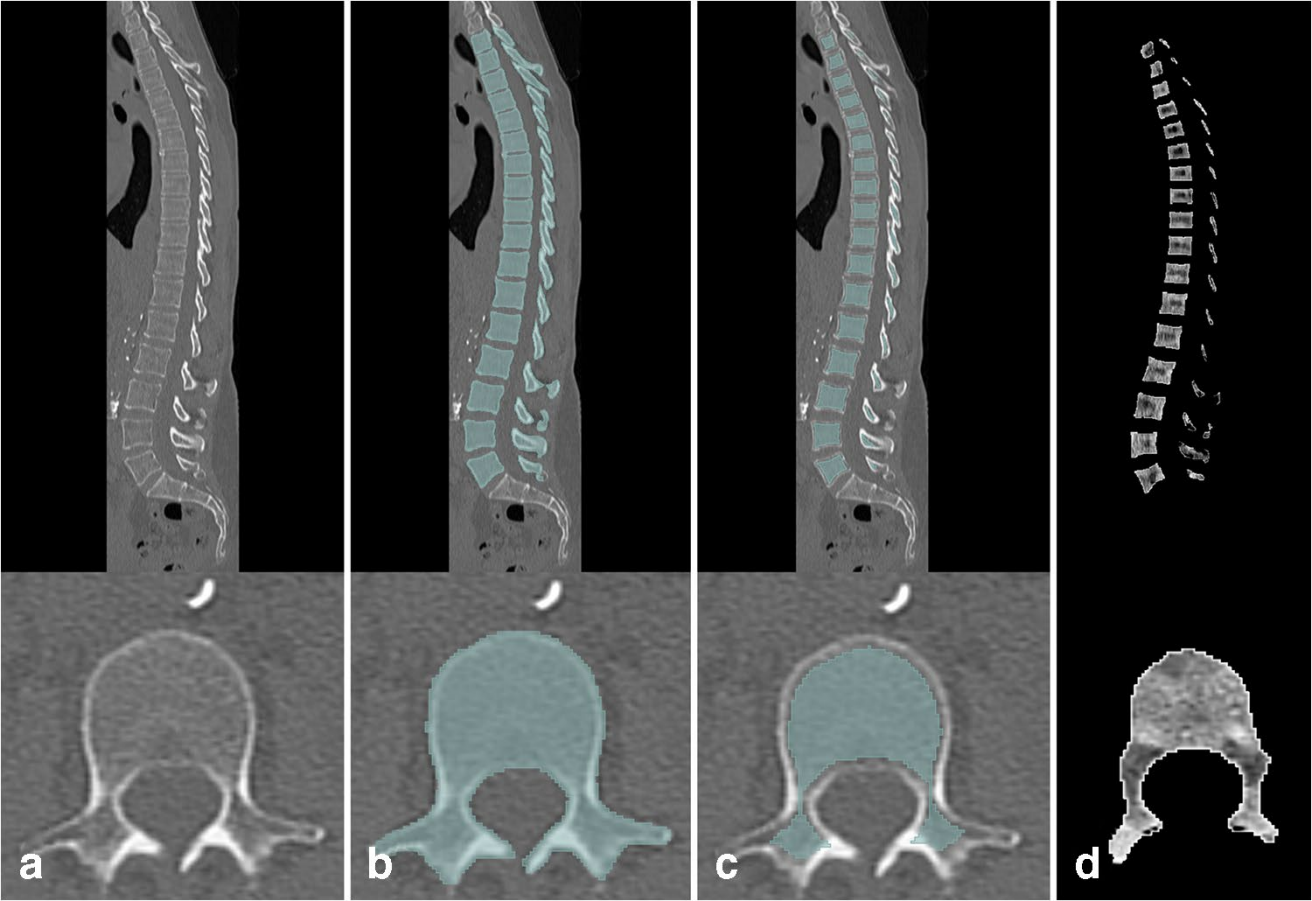
Step-by-step automated segmentation of bone marrow. **a** The monoenergetic CT data served as an input to the pretrained convolutional neural network by Payer et al. **b** The output was limited to 17 vertebrae by a Python script, counting from the most bottom one. Segmentation after the first step included the bordering cortical bone, which was excluded by the “Shrink margin” command of 3D Slicer (**c**). The segmentation was then applied to the virtual non-calcium dataset and used as a mask to obtain a dataset of mere bone marrow attenuation (**d**)

All 35 three-dimensional CT datasets were roughly cropped to a longish cuboid containing the thoracolumbar spine. Automated segmentation of the spine was achieved by a pre-trained convolutional neural network [19, 20]. Seventeen *vertebrae* counting from bottom upwards were marked as a volume of interest (VOI) by a *Python* script. The *SciPy* command *“scipy.ndimage.binary_erosion”* was executed by 3 mm in order to exclude the bordering cortical bone from our VOIs, which does not contain BM (Fig. 1, panel c) [21, 22]. The VOI outlining 17 *vertebrae* was then automatically applied to the VNCa dataset. Our method did not require specific user interaction. Visualization was realized by the open source software *3D Slicer* [23, 24].

A histogram of attenuation (range: − 1024 HU—+ 3071 HU, bin width 5) was extracted from this three-dimensionally segmented VNCa spinal cord section for each patient. BM attenuation on VNCa images was visualized in a histogram for MM and MGUS patients after standardization of the VOI size to 336.0 cm^3^.Batch processing of 35 CT datasets took about 3.5 h on a standard desktop computer (processor: Intel ® Core™ i9-9980HK CPU with 2.4 GHz clock frequency).

### Analysis of the attenuation histograms

After virtual removal of the bone mineral portion during VNCa post-processing, the remaining BM attenuation consists of the fatty and soft-tissue portion, as introduced by the three-compartment model above. In order to estimate the relative amount of fatty and soft-tissue compartments, the number of voxels > 0 HU in the VNCa data was divided by the total volume of the segmented BM, resulting in the non-fatty attenuating portion of BM for each patient. This threshold of 0 HU was adopted from earlier studies for discrimination of physiological and infiltrated BM in MM [9]. The peak of BM attenuation below − 1000 HU apparently resulted from calcium removal from densely calcified structures, e.g., cortical bone or bone islands, which do not make up the BM space. Therefore, for calculation of the total volume of BM, only voxels with attenuation >  − 1000 HU were considered.

### Texture analysis of VNCa BM images

A supplementary radiomics analysis was performed by extracting textural features of the VNCa post-processed BM space, which is illustrated in detail in supplementary data 1 [25].

### Bone mineral density measurements

Bone mineral density (BMD) was quantified by a CE-certified software for phantom-less, in-body calibrated measurements (IntelliSpace, Philips Healthcare) [26–29]. BMD measurements were performed on the first to third lumbar vertebrae, as outlined in the software’s manual. In case of focal osteolytic bone lesions or vertebral fractures, measurements were extended to the lower vertebral spine (*n* = 3 patients). Density measurements in the paravertebral muscles (erector spinae muscle) and the subcutaneous fat tissue served as a spectrometric calibration. BMD measurements were repeated by two independent, blinded radiologists with 3 and 4 years of experience to assess inter-reader agreement.

### Visual assessment of CT data

Conventional CT images were independently screened by three blinded radiologists (3, 3, and 4 years of experience) for myeloma defining osteolytic lesions, as outlined by the IMWG [16]. Each CT was declared positive or negative for myeloma defining osteolytic lesions by majority vote.

### Statistical assessment

Statistical analysis was performed in *R* language for statistical computing, R Foundation, version 4.0.0. Shapiro–Wilk’s test was performed to test the data for normal distribution, using the *R* library *dplyr* [30]. Receiver operating characteristic analysis was carried out by the *R* library *pROC* with the predictor “non-fatty portion of BM in VNCa” and the binary outcomes “evidence of at least one MM defining osteolytic lesion” and “clinical diagnosis of MM by IMWG criteria” [31].

Inter-rater reliability of BMD measurements was reported by the intraclass correlation coefficient (ICC) in a single rater type, two-way random-effects model (ICC2), using the *R* library *irr* (supplementary data 2) [32, 33].

Appropriate sample size of the multivariate regression was calculated by the software G*Power for a desired power level of 0.8, significance level of 0.05, and an estimated medium to large effect size (*f*^2^ = 0.32) of the main independent variable, based on reported data (supplementary data 3) [34, 35].

Statistical significance was defined as *p* ≤ 0.05. Data is stated as mean ± standard deviation, if not otherwise specified.

## Results

**Fig. 2:**
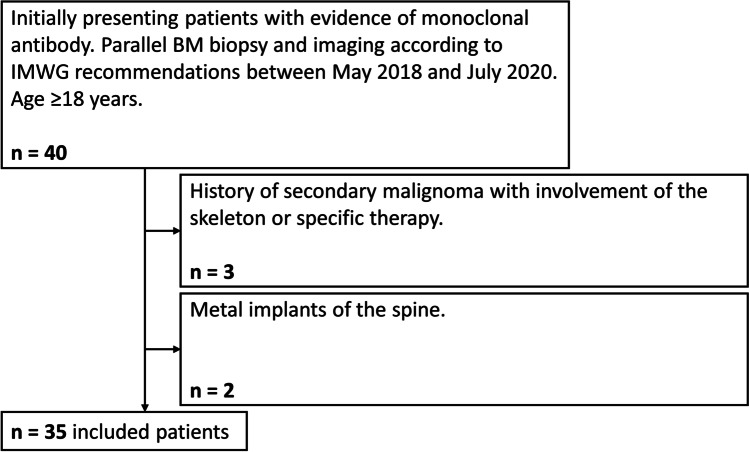
Inclusion chart of the study population

Patient enrollment resulted in a study population of 35 individuals, after exclusion of five patients (Fig. 2). Mean age was 64.6 ± 12.4 years. Eighteen patients were female, and 17 male. Twenty patients initially presented with MM, 14 with MGUS, and one smoldering myeloma. For the subsequent analysis, the patient with smoldering myeloma was included in the MM subgroup, since BM Infiltration was above the MGUS threshold (≥ 10%). BM biopsy and DECT scan were separated by a median of 4.5 days [interquartile range 1.0–12.5 days]. In the MM subset, median BM infiltration was 40% [interquartile range 12.5–70.0] and median BMD was 96.8 mg/ml [interquartile range 89.6–110.4]. Throughout MGUS patients, median BM infiltration was 0% [interquartile range 0–0] and median BMD was 95.6 mg/ml [interquartile range 82.7–106.1]. Patient characteristics are summarized in Table 1.

**Table 1:** Patient characteristics

Observed items	Multiple myeloma/smoldering myeloma	Monoclonal gammopathy of unknown significance
Number	*n* = 21	*n* = 14
Patient age (mean ± standard deviation)	68.2 ± 11.6	59.1 ± 11.9
Patient sex	11 female/10 male	7 female/7 male
Bone marrow infiltration (median, interquartile range)	40% [12.5–70.0]	0% [0–0]
Bone mineral density	96.8 mg/ml [89.6–110.4]	95.6 mg/ml [82.7–106.1]

### Results of automated segmentation

**Fig. 3:**
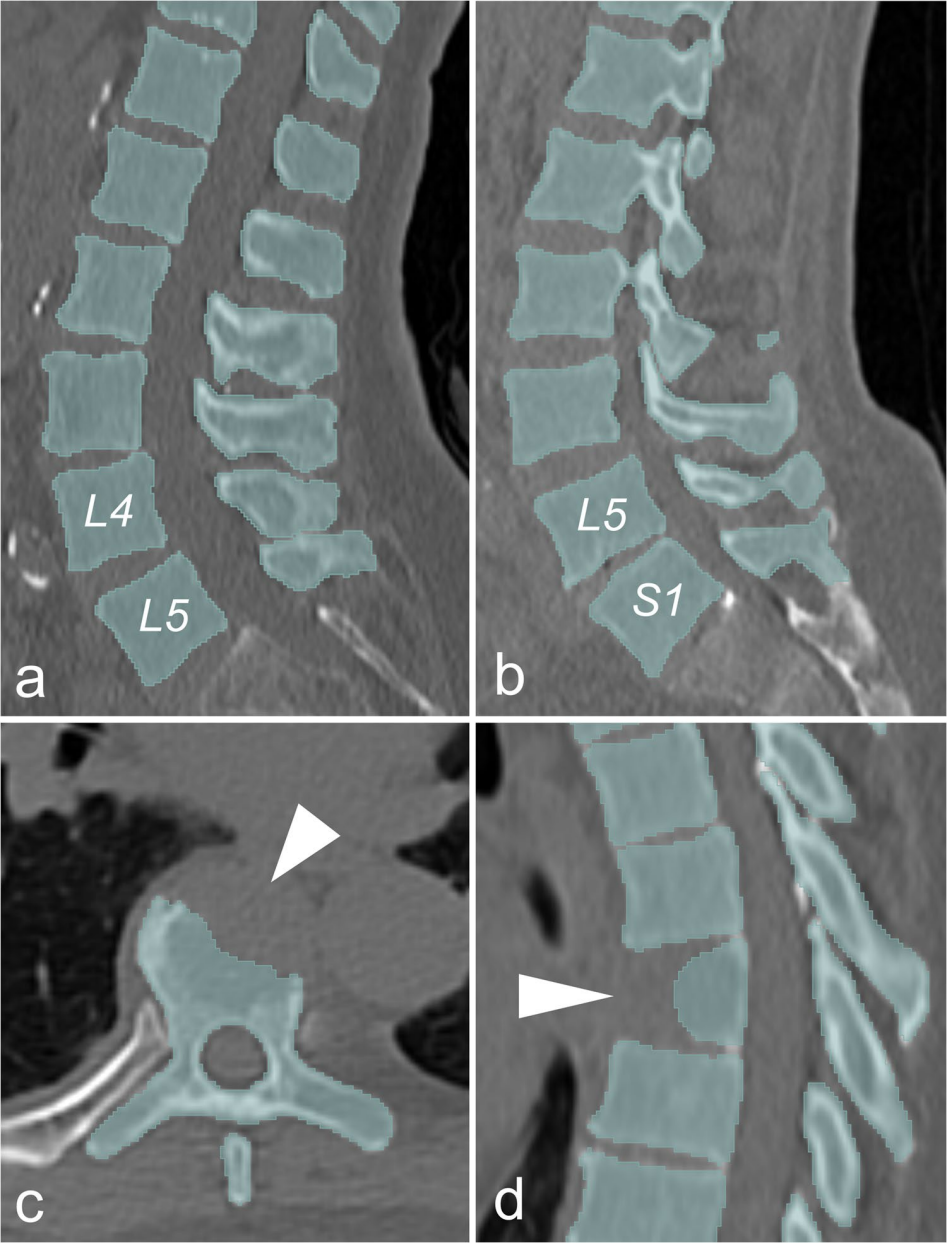
Results of the automated segmentation of the thoracolumbar spine. Exemplary sagittal and axial slices of non-contrast CT with green overlay of AI-supported segmentation of three patients with evidence of monoclonal antibody (**a**, **b** and **c**/**d**). In 34 out of 35 patients, 17 consecutive vertebrae were segmented. In 29 patients the thoracolumbar spine was annotated correctly (**a**). The most common misclassification was the segmentation of Th2-S1 instead of Th1-L5, due to lumbosacral transitional *vertebrae* (four patients, **b**). Segmentation results were not manually altered in order to exclude bias by specific user interaction throughout our automated method. The convolutional neural network by Payer et al. was trained with healthy individuals only. However, osteolytic bone lesions were well respected and spared from the segmentation (white arrowheads, **c**/**d**)

In 34 out of 35 patients, 17 consecutive vertebrae were identified by the neural network and our *Python* algorithm. In one patient, instead of the second thoracic vertebra, the seventh cervical vertebra was segmented. In 29 out of 35 patients, the thoracolumbar spine was segmented anatomically correct (Th1-L5). In four patients, segmentation started at the second thoracic vertebra (Th2-S2), apparently due to lumbosacral transitional vertebrae (Fig. 3). In one case, segmentation started at the seventh cervical vertebra (C7-L4). The segmentation results were not manually corrected, in order to prove feasibility of the automated workflow of our method and enable batch processing. Mean volume of the segmented BM space was 336.0 ± 99.3 cm^3^. Since our study population comprised patients at first presentation, there was no advanced stage of spinal destruction due to myeloma bone disease. Even though the neural network was trained with healthy individuals only, the automated segmentation process respected the present spinal osteolytic lesions (Fig. 3) [19, 20].

### Analysis of the attenuation histograms

**Fig. 4:**
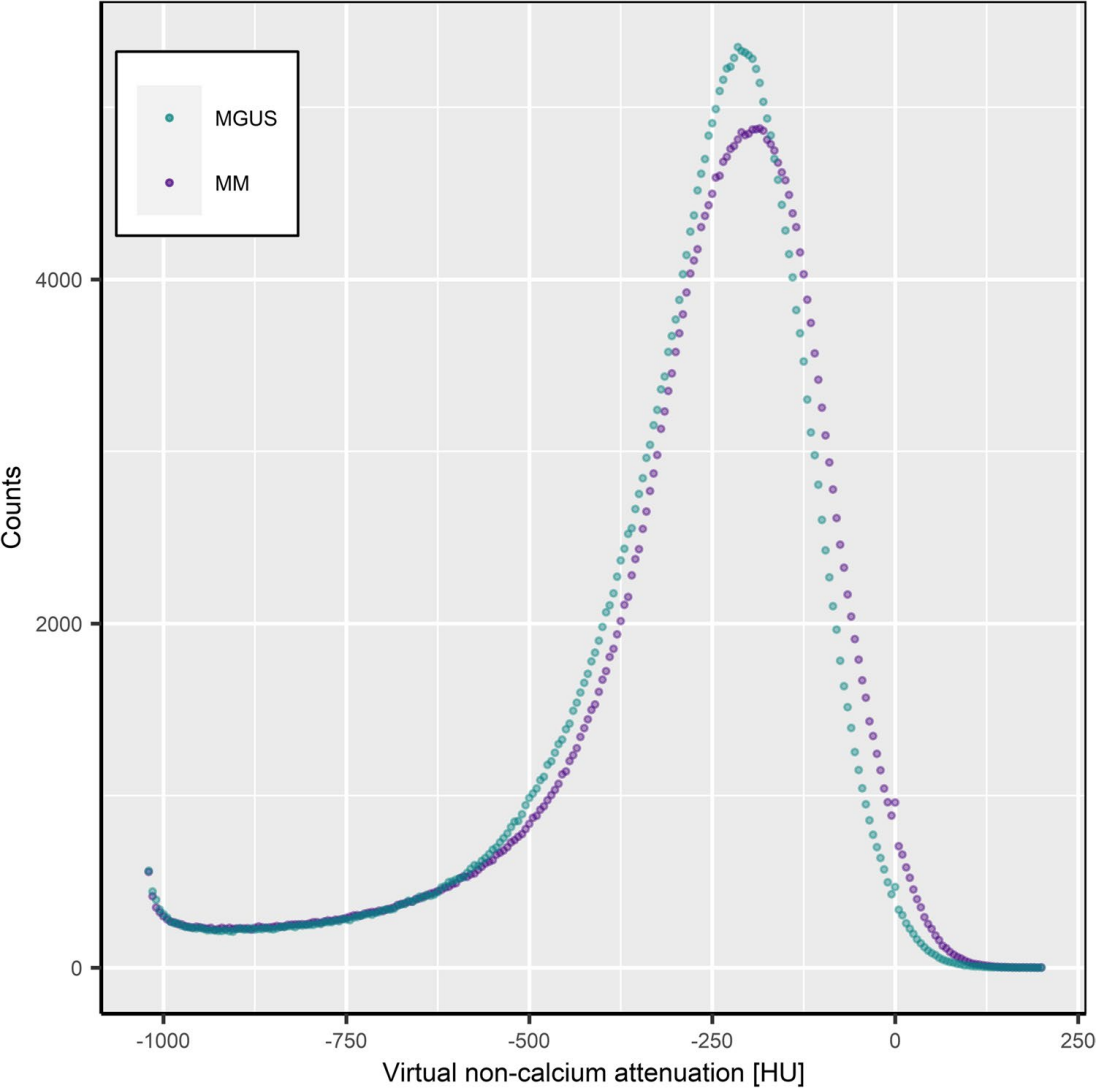
Histogram of bone marrow (BM) attenuation on virtual non-calcium CT images. Voxel counts of smoldering myeloma/multiple myeloma (MM) patients and patients with history of monoclonal gammopathy of unknown significance (MGUS) were plotted to the histogram. Automatically segmented BM was standardized to a volume of 336.0 cm^3^; thus, both plots cover the same area under the curve. The MGUS peak is higher and shifted towards negative attenuation, when compared to the MM peak. The non-fatty portion of the BM > 0 HU was larger throughout MM patients. The peak at the left end of both histograms can be explained by the virtual calcium removal, which results in extreme negative attenuation of structures with dense calcification. Further, the large overlap of both histograms in the range of negative HU values might reflect the resemblance of bone mineral density of both subsets: after virtual calcium removal, the bone mineral density might crucially influence the negative part of the attenuation histogram

The peak of the MGUS histogram was higher (5349 vs. 4876 standardized voxels) and shifted towards negative HU values (− 215 HU vs. − 185 HU), when compared to the MM group (Fig. 4).

**Fig. 5:**
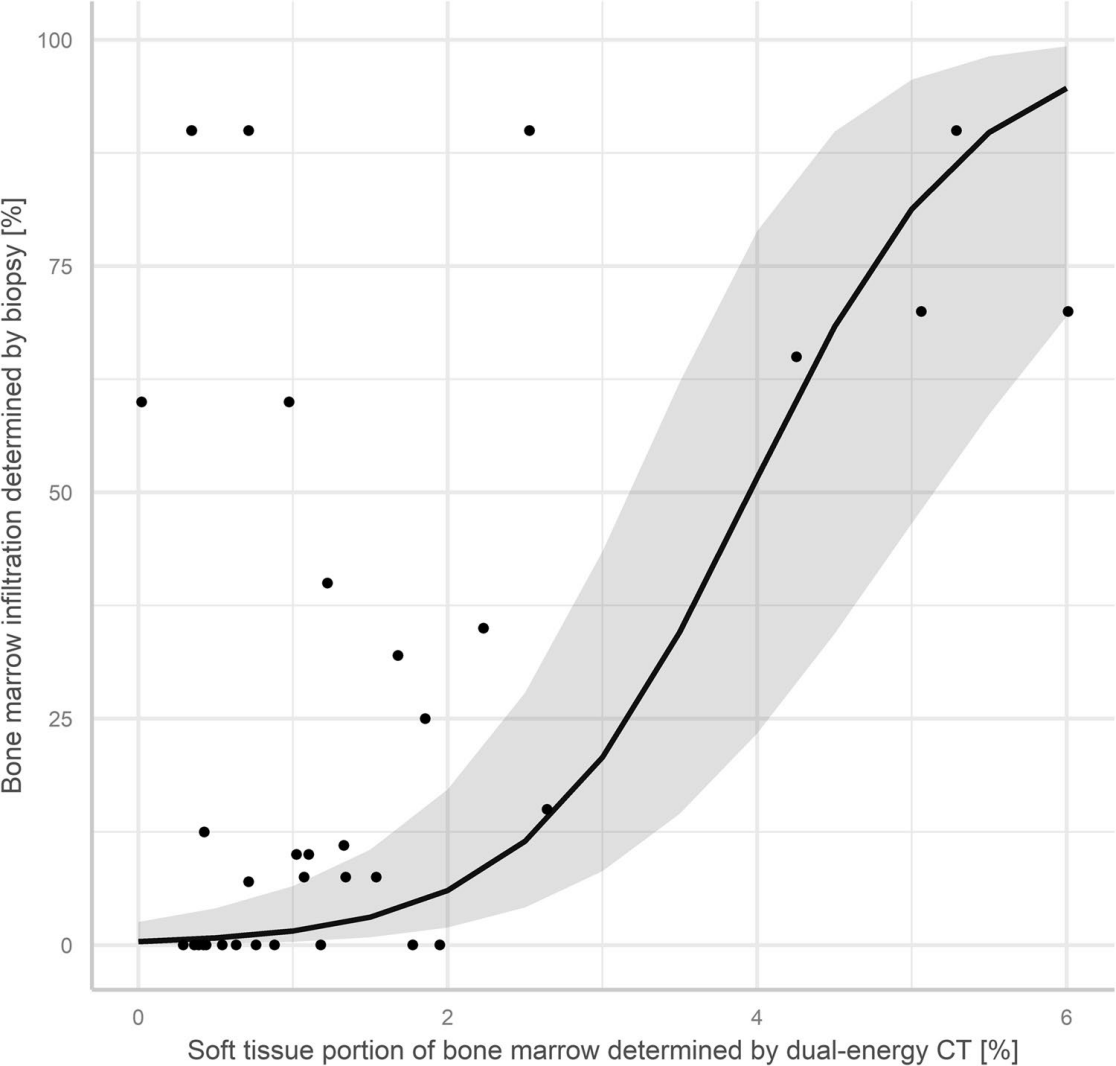
Multivariate regression of the non-fatty portion of bone marrow in virtual non-calcium CT and the biopsy-determined bone marrow infiltration. The association was highly significant (*p* = 0.007) and moderately strong (*r* = 0.46), after the inclusion of bone mineral density as a control variable. The regression line of the model-based analysis is plotted in black with a gray band marking the 95% confidence interval

The non-fatty portion of the BM (> 0 HU) on three-dimensional VNCa data ranged from 0.02 to 6.01%. The median portion of non-fatty BM (> 0 HU) in patients with evidence of osteolytic lesions on conventional CT (*n* = 13) was 1.33% [1.07–4.25%]. The corresponding portion for MM patients without osteolytic lesions (*n* = 7) was 1.18% [0.64–1.89%]. The non-fatty portion of BM in patients without evidence of osteolytic lesions was generally lower; however, this difference remained non-significant (Wilcoxon test, *p* = 0.41). To further investigate the relationship between the non-fatty portion of BM and BM infiltration, a multivariate regression analysis was conducted (Fig. 5). Since it is known that the BMD interacts with the vertebral bone marrow fat content and the BM infiltration in MM, it was included as a control variable [36, 37]. This ensures that the effect of the main independent variable, the non-fatty portion of BM, is not overestimated or driven by BMD. For modelling of the regression, the dependent variable was logit transformed to account for its bounded nature [38]. The regression results showed that non-fatty portion of BM is a significant predictor of the BM infiltration (*p* = 0.007, *r* = 0.46). The effect of the control variable BMD was not significant (*p* = 0.30). Variance inflation factors below 1.5 excluded collinearity among predictor variables in the model [39]. A Breusch-Pagan test indicated the presence of heteroskedasticity in the residuals (*p* < 0.05). This problem has been addressed by the use of robust standard errors according to White [40].

**Fig. 6:**
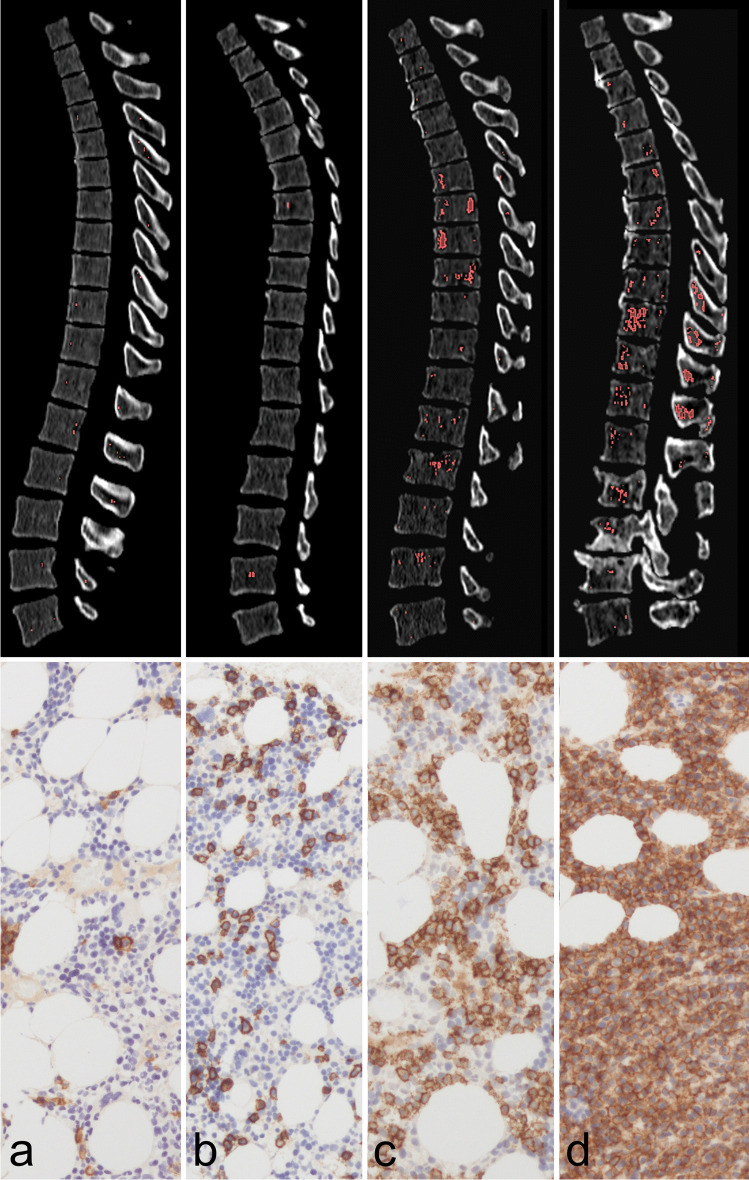
Spine infiltration by multiple myeloma (MM) displaces the fatty bone marrow (BM). Four patients (**a**–**d**) with parallel dual-energy CT (DECT, top row) and CD138-immunostained BM biopsy in 200 × amplification (bottom row) are presented as an example. The thoracolumbar spine was automatically segmented by a convolutional neural network. The red overlay on the DECT slices marks voxels with attenuation > 0 HU in virtual non-calcium (VNCa) post-processed images. Percentage of BM infiltration, as determined by biopsy, was 0–5%, 10–15%, 60–70%, and 90% for patients **a**–**d**, respectively. With rising BM infiltration, an increase of the non-fatty attenuating portion of BM on VNCa images is visually assessable (larger patches of red overlay) and measurable (0.3%, 0.4%, 4.3%, and 5.4% for patients **a**–**d**, respectively). Correspondingly, the histological images demonstrate an expansion of CD138 + , brownish stained plasma cells and a displacement of the translucent, fatty vacuoles. We hypothesize that these histological findings correspond to the raise of attenuation, which we observed on VNCa BM data

The association of the non-fatty portion of BM with the degree of BM infiltration by pathology report is illustrated in Fig. 6.

### Prediction of myeloma defining lesions and the clinical diagnosis of MM by whole-spine BM attenuation

Image interpretation of the conventional CT images by three experienced radiologists found evidence of myeloma defining osteolytic lesions in 13 out of 35 patients. Thirty-one readings resulted in univocal results of the three readers; four cases were determined by majority vote.

We performed a receiver operating characteristic analysis with the predictor “non-fatty portion of BM in VNCa” and the binary outcome “evidence of at least one MM defining osteolytic lesion.” Here, the area under the curve was 0.70 [0.49–0.90]; maximum sensitivity and specificity were 0.85 (11/13) and 0.59 (13/22, Youden), respectively, applying a cutoff of non-fatty BM portion of 0.93%. However, the power level of the ROC analysis for a desired significance level of 0.05 was 0.53, only. Area under the ROC curve for prediction of the “clinical diagnosis of MM” by the “non-fatty portion of BM in VNCa” was 0.71 [0.54–0.89]. Here, the power for a desired significance level of 0.05 was 0.59. The best threshold by Youden’s method was again 0.93%. From patients that did not show myeloma defining bone lesions on conventional CT images, but who were clinically diagnosed with MM (*n* = 8), an above threshold non-fatty portion of BM in DECT could identify five (> 0.93, sensitivity 0.63, 5/8, specificity 0.71, 10/14).

## Discussion

In order to restrict BM biopsy in patients with evidence of monoclonal antibody to the essential and obligatory minimum, we investigated VNCa imaging in DECT to non-invasively estimate BM infiltration. Automated segmentation of the BM space yielded visually excellent results. A subsequent, histogram-based analysis was capable to demonstrate a positive association between the portion of non-fatty attenuation on VNCa images and the biopsy-determined BM infiltration, remaining significant after adjusting for BMD as a control variable (*p* = 0.007, *r* = 0.46). BMD was not significantly different between the MM and MGUS subset, which we account to the early timepoint of assessment of therapy naïve patients without advanced osteolysis (Wilcoxon test, *p* = 0.49). Introducing a fully automated solution, our approach offers possibilities for clinical use without depleting resources for manual spine segmentation or observation by trained personal. Since DECT datasets were obtained according to IMWG guidelines, the presented technique does not require higher economic cost or radiation exposure [16].

Non-invasive estimation of BM infiltration might not only help to avoid unnecessary BM biopsy of MGUS patients, but also validate BM biopsy of MM patients, which is prone to sampling error due to the patchy morphology of MM bone infiltration [41]. While a typical BM biopsy by a 15G needle obtains a 1-cm, cylindrical tissue sample of approximately 0.003 cm^3^, the average volume of BM space that was analyzed in our approach is 336.0 cm^3^, which might be more representative of the BM infiltration status. BM biopsy is further biased by practical limitations, such as operator experience, which is most likely ruled out by the automated method of our study [42].

Further, we observed a trend that an above threshold portion of non-fatty BM might preselect patients with higher pre-test probability of myeloma defining bone lesions and clinical diagnosis of MM. This part of our analysis, however, lacked the desired statistical power level due to a limited sample size.

A recent study correlated BM texture analysis in dual-energy CT (DECT) with BM infiltration in MM [34]. The study extracted 92 *PyRadiomics* features for each of 56 patients, which yielded six significant features for Pearson’s correlation with BM infiltration. In our study, the same 92 features were calculated in a supplementary analysis (supplementary data 1), resulting in two significant descriptors. However, only one descriptor was significant in both studies (*“ngtdm_Contrast”*), which demonstrates the common problem of reproducibility and generalizability for radiomics studies with inappropriately small sample sizes [43, 44]. On the other hand, our data suggest a significant association, which was investigated hypothesis-driven and based on biological causalities. We observed a decreasing portion of fatty BM in contrast to an increasing fluid-like and soft tissue attenuation of BM in MM, which is well-known on a microscopic level, since in MM fatty BM is displaced by plasma cells [45]. Our observation is in line with recent literature, suggesting VNCa imaging to estimate the degree of displacement of fatty BM in MM [13].

There are some limitations that need to be discussed. First, our cutoff for discrimination of pathologic BM was defined at 0 HU, which is motivated by the hypothesis that healthy, fatty BM is displaced by soft tissue– and fluid-like attenuating infiltration. A recent study found a similar cutoff between − 3 HU and 4 HU [9], while another research proposed − 44.9 HU for identification of infiltrated BM in MM [12]. The authors claim that the discrepancy most likely arises due to technical aspects [12]. However, both studies relied on subjective and semi-objective (manual region of interest measurements) analysis of the VNCa images. Since VNCa post-processing is still not regularly established in clinical practice, most radiologists have limited experience in assessment of VNCa data, which might introduce an inter-reader bias. Hence, our quantitative results are limited to the specific scanner and reconstruction used, while the general methodology is considered reproducible. In this context, a multi-center, multi-vendor study would be of interest, yet, was out of scope of this investigation. The multivariate regression plot presents several outliers in the top left quadrant, which precluded prediction of absolute changes in biopsy-determined BM infiltration per increase of BM attenuation. To elaborate on the highly significant regression, a larger study population might allow to limit its confidence intervals for absolute predictions.

Our AI-supported algorithm segmented the spine into 17 *vertebrae* in all cases, while some misclassifications occurred as described above. Since MM is a systemic disease and we did not expect a significant bias by incorrect anatomic numeration of the vertebrae (e.g., segmentation of T2-S1 instead of T1-L5 in case of a lumbosacral transitional *vertebra*), there was no necessity to interrupt the batch processing for manual alteration. Last, we included a rather small number of patients as we wanted to study treatment-naïve patients with concurrent BM biopsy, only. Hence, our feasibility study does not allow for specific conclusions about the subset of smoldering myeloma patients, since only a single case was examined. Further, performance of the threshold-based analysis for identification of MM in patients with negative conventional CT was poor (sensitivity 0.63, specificity 0.71), possibly outlining a limitation of our method to assess subtle, non-lytic BM infiltration; however, final evaluation requires a larger sample size for further examination.

Concluding, our work demonstrates the feasibility of an automated, AI-supported method to non-invasively estimate the degree of BM infiltration in MM and its premalignant conditions. In line with the recent clinical trend to question BM biopsy at first presentation of patients with evidence of monoclonal protein, we propose a tool for clinical decision support to avoid unnecessary invasive BM diagnostic.

## Supplementary Information

Below is the link to the electronic supplementary material.

Supplementary file1 (DOCX 80 KB)

## Abbreviations

AI: Artificial intelligence
BM: Bone marrow
BMD: Bone mineral density
DECT: Dual-energy computed tomography
IMWG: International Myeloma Working Group
MGUS: Monoclonal gammopathy of unknown significance
MM: Multiple myeloma
VNCa: Virtual non-calcium
VOI: Volume of interest

## Declarations

### Guarantor

The scientific guarantor of this publication is Philipp Fervers.

### Statistics and biometry

One of the authors has significant statistical expertise.

### Methodology

• retrospective

• observational study

• performed at one institution

## References

[1] Ho M, Patel A, Goh CY (2020). Changing paradigms in diagnosis and treatment of monoclonal gammopathy of undetermined significance (MGUS) and smoldering multiple myeloma (SMM). Leukemia.

[2] Kazandjian D (2016). Multiple myeloma epidemiology and survival, a unique malignancy. Semin Oncol.

[3] Mateos MV, Landgren O (2016) MGUS and Smoldering Multiple Myeloma: Diagnosis and Epidemiology. In: Roccaro A., Ghobrial I. (eds) Plasma Cell Dyscrasias. Cancer Treatment and Research, vol 169. Springer, Cham. 10.1007/978-3-319-40320-5_1 10.1007/978-3-319-40320-5_1 27696254

[4] Landgren O, Kyle RA, Rajkumar SV (2011). From myeloma precursor disease to multiple myeloma: new diagnostic concepts and opportunities for early intervention. Clin Cancer Res.

[5] Sidiqi MH, Aljama M, Kumar SK (2020). The role of bone marrow biopsy in patients with plasma cell disorders: should all patients with a monoclonal protein be biopsied?. Blood Cancer J.

[6] Hjortholm N, Jaddini E, Hałaburda K, Snarski E (2013). Strategies of pain reduction during the bone marrow biopsy. Ann Hematol.

[7] Tschautscher MA, Jevremovic D, Buadi FK (2020). Utility of repeating bone marrow biopsy for confirmation of complete response in multiple myeloma. Blood Cancer J.

[8] Abdullayev N, Große Hokamp N, Lennartz S (2019). Improvements of diagnostic accuracy and visualization of vertebral metastasis using multi-level virtual non-calcium reconstructions from dual-layer spectral detector computed tomography. Eur Radiol.

[9] Thomas C, Schabel C, Krauss B et al (2015) Dual-energy CT: virtual calcium subtraction for assessment of bone marrow involvement of the spine in multiple myeloma. AJR Am J Roentgenol 204(3):W324–31. 10.2214/AJR.14.12613 10.2214/AJR.14.12613 25714318

[10] Pache G, Krauss B, Strohm P (2010). Dual-energy CT virtual noncalcium technique: detecting posttraumatic bone marrow lesions - feasibility study. Radiology.

[11] Burke MC, Garg A, Youngner JM (2019). Initial experience with dual-energy computed tomography-guided bone biopsies of bone lesions that are occult on monoenergetic CT. Skeletal Radiol.

[12] Kosmala A, Weng AM, Heidemeier A (2018). Multiple myeloma and dual- energy CT: diagnostic accuracy of virtual noncalcium technique for detection of bone marrow infiltration of the spine and pelvis. Radiology.

[13] Kosmala A, Weng AM, Krauss B (2018). Dual-energy CT of the bone marrow in multiple myeloma: diagnostic accuracy for quantitative differentiation of infiltration patterns. Eur Radiol.

[14] Palmer WE, Simeone FJ (2018). Can dual-energy CT challenge MR imaging in the diagnosis of focal infiltrative bone marrow lesions?. Radiology.

[15] Fervers P, Glauner A, Gertz R (2021). Virtual calcium-suppression in dual energy computed tomography predicts metabolic activity of focal MM lesions as determined by fluorodeoxyglucose positron-emission-tomography. Eur J Radiol.

[16] Moulopoulos LA, Koutoulidis V, Hillengass J (2018). Recommendations for acquisition, interpretation and reporting of whole body low dose CT in patients with multiple myeloma and other plasma cell disorders: a report of the IMWG Bone Working Group. Blood Cancer J.

[17] Hokamp NG, Maintz D, Shapira N (2020). Technical background of a novel detector-based approach to dual-energy computed tomography. Diagnostic Interv Radiol.

[18] Neuhaus V, Lennartz S, Abdullayev N (2018). Bone marrow edema in traumatic vertebral compression fractures: diagnostic accuracy of dual-layer detector CT using calcium suppressed images. Eur J Radiol.

[19] Sekuboyina A, Bayat A, Husseini ME et al (2020) VerSe: a Vertebrae labelling and segmentation benchmark for multi-detector CT images. Medical Image Analysis 73(2021):102166. 10.1016/j.media.2021.102166 10.1016/j.media.2021.102166 34340104

[20] Payer C, Štern D, Bischof H, Urschler M (2020) Coarse to fine vertebrae localization and segmentation with spatialconfiguration-Net and U-Net. In: Proceedings of the 15th International Joint Conference on Computer Vision, Imaging and Computer Graphics Theory and Applications. SCITEPRESS - Science and Technology Publications, 124–133

[21] Virtanen P, Gommers R, Oliphant TE et al (2020) SciPy 1.0: fundamental algorithms for scientific computing in Python. Nat Methods 17:261–272. 10.1038/s41592-019-0686-2 10.1038/s41592-019-0686-2 PMC7056644 32015543

[22] Harris CR, Millman KJ, van der Walt SJ (2020). Array programming with NumPy. Nature.

[23] 3D Slicer. https://www.slicer.org/. Accessed 13 Jan 2021

[24] Kikinis R, Pieper SD, Vosburgh KG (2014) 3D slicer: a platform for subject-specific image analysis, visualization, and clinical support. In: Jolesz F. (eds) Intraoperative Imaging and Image-Guided Therapy. Springer, New York, pp. 277–289. 10.1007/978-1-4614-7657-3_19

[25] Van Griethuysen JJM, Fedorov A, Parmar C (2017). Computational radiomics system to decode the radiographic phenotype. Cancer Res.

[26] Kottlors J, Große Hokamp N, Fervers P (2021). Early extrapulmonary prognostic features in chest computed tomography in COVID-19 pneumonia: bone mineral density is a relevant predictor for the clinical outcome - A multicenter feasibility study. Bone.

[27] Boden SD, Goodenough DJ, Stockham CD (1989). Precise measurement of vertebral bone density using computed tomography without the use of an external reference phantom. J Digit Imaging.

[28] Neuhaus V, Abdullayev N, Hellmich M (2016). Association of quality and quantity of bone metastases and computed tomography volumetric bone mineral density with prevalence of vertebral fractures in breast cancer patients. Clin Breast Cancer.

[29] Mueller DK, Kutscherenko A, Bartel H (2011). Phantom-less QCT BMD system as screening tool for osteoporosis without additional radiation. Eur J Radiol.

[30] Wickham H, François R, Henry L, Müller K (2018). dplyr: a grammar of data manipulation. R package version 1.0.7. https://CRAN.R-project.org/package=dplyr

[31] Robin X, Turck N, Hainard A (2011). pROC: An open-source package for R and S+ to analyze and compare ROC curves. BMC Bioinformatics.

[32] Koo TK, Li MY (2016). A guideline of selecting and reporting intraclass correlation coefficients for reliability research. J Chiropr Med.

[33] Gamer M, Lemon J, Fellows I, Singh P (2019) Package “irr”, version 0.84.1: various coefficients of interrater reliability and agreement. https://www.r-project.org

[34] Reinert CP, Krieg E, Esser M,et al (2020) Role of computed tomography texture analysis using dual-energy-based bone marrow imaging for multiple myeloma characterization: comparison with histology and established serologic parameters. Eur Radiol 31(4):2357–2367. 10.1007/s00330-020-07320-8 10.1007/s00330-020-07320-8 PMC7979667 33011876

[35] Faul F, Erdfelder E, Lang AG et al. G*Power 3: A flexible statistical power analysis program for the social, behavioral, and biomedical sciences. Behavior Research Methods 39, 175–191 (2007). 10.3758/BF03193146 10.3758/bf03193146 17695343

[36] Sheu Y, Amati F, Schwartz AV (2017). Vertebral bone marrow fat, bone mineral density and diabetes: the Osteoporotic Fractures in Men (MrOS) study. Bone.

[37] Muchtar E, Dagan A, Robenshtok E (2017). Bone mineral density utilization in patients with newly diagnosed multiple myeloma. Hematol Oncol.

[38] Baum CF (2008). Stata tip 63: Modeling proportions. Stata J.

[39] Chatterjee S, Hadi AS (2012) Regression analysis by example, 5th edn. Wiley, Hoboken, New Jersey

[40] White H (1980). A heteroskedasticity-consistent covariance matrix estimator and a direct test for heteroskedasticity. Econometrica.

[41] Blebea JS, Houseni M, Torigian DA (2007). Structural and functional imaging of normal bone marrow and evaluation of its age-related changes. Semin Nucl Med.

[42] Marinelli LM, Fang H, Howard MT (2018). Bone marrow biopsy operator experience and impact on aspirate, biopsy, and ancillary testing quality. Mayo Clin Proc Innov Qual Outcomes.

[43] Park JE, Park SY, Kim HJ, Kim HS (2019). Reproducibility and generalizability in radiomics modeling: possible strategies in radiologic and statistical perspectives. Korean J Radiol.

[44] Lambin P, Leijenaar RTH, Deist TM (2017). Radiomics: the bridge between medical imaging and personalized medicine. Nat Rev Clin Oncol.

[45] Dutoit JC, Verstraete KL (2017). Whole-body MRI, dynamic contrast-enhanced MRI, and diffusion-weighted imaging for the staging of multiple myeloma. Skeletal Radiol.

