# Supplementary material for: Feasibility of artificial intelligence–supported assessment of bone marrow infiltration using dual-energy computed tomography in patients with evidence of monoclonal protein — a retrospective observational study
Source: Eur Radiol. 2021 Dec 18;32(5):2901–11. doi: 10.1007/s00330-021-08419-2 (PMC9038860; doi:10.1007/s00330-021-08419-2)
Supplement: Supplementary file 1 — Supplementary file1 (DOCX 80 KB) [file 330_2021_8419_MOESM1_ESM.docx]

**Supplementary Material**

**Supplementary Data 1: Analysis of PyRadiomics features**

Textural features were defined by the open-source library *PyRadiomics*, release 3.0.1 (1). We refrained from extracting the 14 *PyRadiomics* “shape descriptors”, since a correlation of the shape of the segmented spine section and BM infiltration was not expected. After extraction from the automatically segmented VNCa BM data, each of the 92 extracted *PyRadiomics* descriptors was correlated with the BM infiltration as determined by BM biopsy. For two descriptors the correlation was significant (p<0.05), which is summarized in supplementary table 1.

**Supplementary Tab. 1. PyRadiomics features, which correlate significantly with bone marrow (BM) infiltration as determined by biopsy.**

| p | Pearson’s r | Name of descriptor |
| --- | --- | --- |
| *0.04* | *0.35* | *glcm_ClusterProminence* |
| *<0.05* | *0.34* | *ngtdm_Contrast* |

Two out of 92 Pyradiomics descriptors correlated significantly with the BM infiltration.

**Supplementary Data 2: Intra- and inter-reader agreement of BMD measurements.**

BMD measurements were repeated on a subset of n=10 patients by two radiologists (three and four years of experience). Further, one radiologist repeated the same BMD measurements one month after the first reading. Intra- and inter-observer variability were reported by the intraclass correlation coefficient (ICC), using the R library *irr*: Various Coefficients of Interrater Reliability and Agreement [1, 2]. Intra- and inter-observer variability were obtained by a two-way random-effects model (ICC2) for single ratings, respectively. Intra-observer variability analysis of BMD measurements yielded an ICC2=0.98, while inter-observer variability was represented by an ICC2=0.99 (Table 1).

Both intra- and inter-observer reliability were excellent (ICC >0.90).

**Supplementary Tab. 2: Intra- and inter-reader agreement of BMD measurements.**

| Reader 1,  Read 1  [mg/ml] | Reader 1,  Read 2  [mg/ml] | Reader 2  [mg/ml] |
| --- | --- | --- |
| 89.6 | 90.2 | 82.6 |
| 99.3 | 99.5 | 101 |
| 146.7 | 146.5 | 156.2 |
| 93.7 | 96.7 | 93.8 |
| 84.2 | 86.5 | 88.8 |
| 96.8 | 92.7 | 93.6 |
| 92 | 91.7 | 90.8 |
| 143.5 | 139.3 | 140.4 |
| 71.9 | 61.2 | 61.6 |
| 101.8 | 99.1 | 95 |

**Supplementary Data 3: Sample size estimation in G*Power.**

The required sample size to ensure adequate statistical power of our analysis was calculated a priori using the software G*Power [3]. Input parameters for sample size estimation were set as follows:

1. **Effect size f² = 0.32** (“medium to large” effect size, which is based on a reported Pearson’s r=0.49 for prediction of BM infiltration by VNCa data and the formula for calculation of *f² = r²/(1-r²)* )[4].
2. **Alpha error probability = 0.05** (required significance level)
3. **Power = 0.80** (typically desired power level)
4. **Number of predictors = 2** (the multivariate regression model includes the main independent variable and BMD as a control variable)

The required sample size was estimated as n = 34 patients (supplementary figure 1).


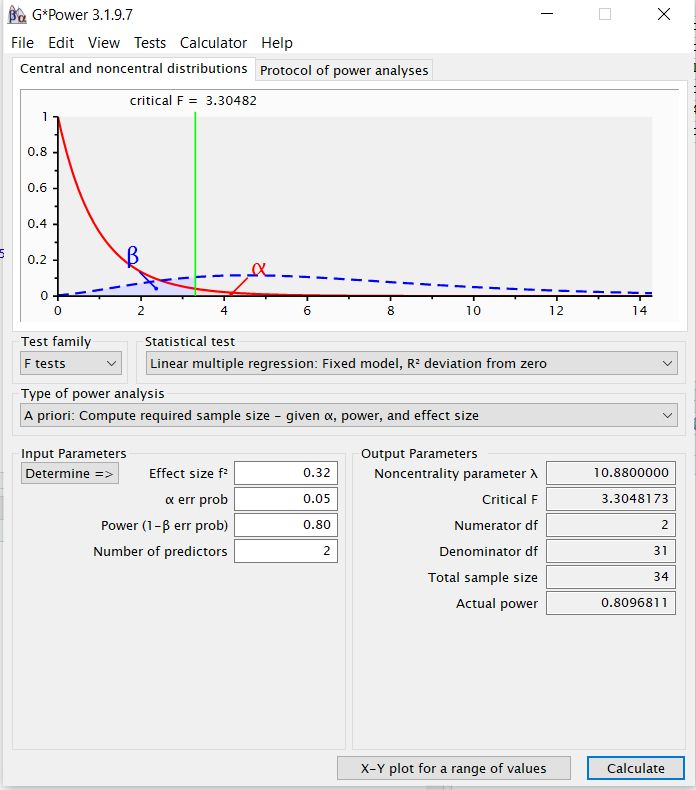


**Supplementary Fig. 1: Sample size estimation in G*Power.**

**References**

1. Koo TK, Li MY (2016) A Guideline of Selecting and Reporting Intraclass Correlation Coefficients for Reliability Research. J Chiropr Med 15:155–163. https://doi.org/10.1016/j.jcm.2016.02.012

2. Matthias Gamer A, Matthias Gamer M (2019) Package “irr” Title Various Coefficients of Interrater Reliability and Agreement

3. Faul F, Erdfelder E, Lang AG, Buchner A (2007) G*Power 3: A flexible statistical power analysis program for the social, behavioral, and biomedical sciences. In: Behavior Research Methods. Psychonomic Society Inc., pp 175–191

4. Reinert CP, Krieg E, Esser M, et al (2020) Role of computed tomography texture analysis using dual-energy-based bone marrow imaging for multiple myeloma characterization: comparison with histology and established serologic parameters. Eur Radiol 1–11. https://doi.org/10.1007/s00330-020-07320-8

5. Van Griethuysen JJM, Fedorov A, Parmar C, et al (2017) Computational radiomics system to decode the radiographic phenotype. Cancer Res 77:e104–e107. https://doi.org/10.1158/0008-5472.CAN-17-0339
